# The impact of patient weight status on healthcare students' attitudes: Two experimental studies in midwifery and dental students

**DOI:** 10.1111/bjhp.70116

**Published:** 2026-10-28

**Authors:** Ankita Sehrawat, Ayse Aslan, Eleanor L. Aldam, Chantelle Wood, Nicola J. Buckland

**Affiliations:** ^1^ School of Psychology University of Sheffield, ICOSS Building Sheffield UK

**Keywords:** dentists, experiment, healthcare, midwives, weight stigma

## Abstract

**Background:**

Weight stigma refers to prejudice and discrimination based on body weight. Healthcare professionals often hold negative attitudes towards patients with overweight, which can negatively affect patient care and health outcomes. However, weight stigma is yet to be explored in midwifery and dental students.

**Methods:**

Two online experimental studies using hypothetical patient vignettes were conducted in midwifery (*n* = 281; Study 1) and dental and non‐dental students (*n* = 528; Study 2). Participants were randomly assigned to view a healthy‐weight, overweight or obese patient vignette and then reported their attitudes, predicted time, advice they would provide and beliefs about obesity. Data were analysed using ANOVA and logistic regression.

**Results:**

In Study 1, midwifery students held significantly more stigmatizing attitudes towards patients who were overweight than healthy‐weight patients; however, there was no significant difference in attitudes towards patients with obesity. Predicted time spent with patients did not significantly differ as a function of patient weight status. In Study 2, dental and non‐dental students reported more stigmatizing attitudes towards overweight and obese patients compared to healthy‐weight patients. Predicted time spent with patients did not differ significantly. Dental and non‐dental students were more likely to offer lifestyle‐related advice to overweight and obese patients. There was no significant relationship between beliefs about obesity and attitudes towards treating patients.

**Conclusions:**

Weight stigma is evident among healthcare students and can affect healthcare advice. Increased awareness of potential biases in midwives and dental students is needed and should form formal training to improve equitable healthcare delivery.

**Statement of contribution**

**What is already known?**

- Weight stigma, including negative attitudes towards people with a higher weight, is found among healthcare professionals, including medical students, nurses and dietitians.
- Weight stigma can negatively impact the quality of care provided. For example, healthcare professionals may conduct fewer diagnostic assessments and spend less time in consultations.
- Experiences of weight stigma can result in patients delaying or avoiding healthcare appointments.

**What does this study add?**

- Our experimental findings confirm that weight stigma extends to midwifery and dental students.
- Midwifery and dental students reported more negative attitudes towards treating patients with a higher weight.
- Dental students were more likely to provide lifestyle‐related advice (considered stigmatizing) to patients with a higher weight than to lower‐weight patients.
- The findings highlight the need for interventions to reduce weight stigma within midwifery and dental settings to support non‐stigmatizing, compassionate and equitable care.

## INTRODUCTION

The prevalence of obesity tripled worldwide between 1975 and 2022, making it one of the most critical public health issues (NCD Risk Factor Collaboration, 2024). Approximately 1 billion people live with obesity, including 880 million adults and 159 million children and adolescents (NCD Risk Factor Collaboration, 2024). Obesity is associated with increased risk of serious comorbidities and mortality, including cardiovascular diseases, type 2 diabetes mellitus, cancers, kidney issues and shorter life expectancy (Berrington de Gonzalez, 2011; Kloock et al., 2023; Wiedemann et al., 2025; World Health Organization, 2025). Effective healthcare services for people with overweight and obesity are therefore important (Kirk & Penney, 2013). However, growing research indicates that weight stigma in healthcare settings can influence patients' health‐related experiences and behaviours.

Weight stigma refers to discriminatory acts and ideologies targeted towards individuals because of their weight and size (World Obesity Federation, 2023). Recent systematic reviews and meta‐analyses have demonstrated that weight stigma has a negative impact on both physical health (Romano et al., 2023) and mental health (Emmer et al., 2020). Contrary to expectations, health professionals, including those who specialize in obesity, can perpetuate weight stigma (Puhl et al., 2016). Within healthcare, overweight patients report receiving inappropriate comments, inadequate treatment and that their concerns are not taken seriously (Brown et al., 2006; Raves et al., 2016). Studies have also reported that as patient weight increases, physicians conduct fewer diagnostic assessments, have shorter consultation durations, give more weight‐related advice and report diminished patience (Hebl & Xu, 2001; Phelan et al., 2015; Rathbone et al., 2023). In turn, weight stigma within healthcare settings has been linked to patients delaying or avoiding healthcare appointments, including cancer screenings (Aldrich & Hackley, 2010; Gnade et al., 2020; Graham et al., 2022).

Weight stigma has also been demonstrated to occur across a range of health professions: negative attitudes towards people with obesity have been reported in exercise and nutrition professionals (Cebioğlu et al., 2022; Jung et al., 2015; Panza et al., 2018), physiotherapists (Setchell et al., 2014), physicians (Sabin et al., 2012), nurses (Robstad et al., 2019) and psychologists (Philip et al., 2025).

However, to date, there is limited published research focusing on trainee professionals' attitudes towards patients with overweight or obesity and how this might impact future behaviour and treatment plans. It is critical to understand the prevalence and impact of weight stigma in future healthcare professionals, particularly in under‐researched fields. For example, pregnant and postpartum individuals with higher body weight frequently report experiencing weight bias from perinatal healthcare providers (DeJoy et al., 2016; Nagpal, da Silva, et al., 2021). However, few studies have measured weight bias among midwifery students as compared to working midwifery professionals (see Robins et al., 2025 for a review). Those studies that do exist have largely focused on assessing explicit and implicit weight stigma among midwifery students (e.g., Jayawickrama et al., 2023; Mulherin et al., 2013), rather than investigating how these attitudes may translate into healthcare delivery. Health organizations have also highlighted the need to address weight bias among perinatal care providers. For example, the 2020 National Academy of Medicine report on birth settings recognized weight bias as a contributor to adverse reproductive health outcomes (Backes & Scrimshaw, 2020).

Dentistry is another health profession where further research on weight stigma is needed. Dietary factors are closely linked to dental issues, such as caries and decay (Mobley et al., 2009; Moynihan & Kelly, 2014). While there is no evidence that obesity and dental caries are linked (Kantovitz et al., 2006; Silva et al., 2013), the influence of diet on dental health may lead trainee dentists to hold preconceived biases towards people with overweight and obesity that could affect care. Understanding whether weight stigma is present in dental care is also important because there is potential for weight management interventions to be delivered in dental settings (Large et al., 2024). However, negative attitudes held by dental professionals and patients' associated experiences of weight stigma are main barriers to implementing such services within dental care (Large et al., 2024). While there has been some research investigating weight stigma in dental settings (see review by Large et al., 2024; Awan et al., 2016; Magliocca et al., 2005), previous research is limited as it has focused solely on attitudes, beliefs and knowledge about obesity, without assessing dental students' behavioural intentions or expected treatment.

Furthermore, the majority of evidence on the consequences of weight stigma in health professionals is derived from correlational or qualitative designs, which limits the ability to draw causal conclusions regarding the impact of weight stigma on clinical attitudes and decision‐making. As highlighted in reviews by Panza et al. (2018) and Philip et al. (2025), there is a clear need for experimental research to more rigorously examine how weight stigma among healthcare professionals and trainees influences clinical judgements and patient‐related outcomes. One of the few studies to adopt an experimental approach is Hebl and Xu (2001). Their research examined the effect of patients' weight status on physicians' attitudes, predicted time spent with patients and diagnostic tests recommended. In an online vignette‐based experiment, physicians were presented with patients experiencing the same health condition (migraine), with patient weight experimentally manipulated as average weight, overweight or obese. The findings indicated that physicians held significantly more negative attitudes towards heavier patients compared to average‐weight patients, reported that they would spend less time with them, and recommended a greater number of diagnostic tests. Notably, these additional tests were largely focused on weight‐related issues rather than the primary presenting complaint. Physicians rated heavier patients as significantly less capable of self‐care and lacking discipline. These findings align with broader evidence showing that healthcare providers who attribute obesity to individual‐level factors (e.g., lack of willpower, unhealthy eating and physical inactivity) are more likely to endorse negative attitudes towards higher‐weight patients (Miller Jr et al., 2013; Pantenburg et al., 2012) and more likely to recommend weight‐based behavioural changes, such as diet and weight management (Bleich et al., 2013; Ogden & Jubb, 2008). More recently, another experimental study showed that medical students reported greater discomfort and avoidance, expected greater concealment and fear of judgement and recalled symptoms less accurately for patients with obesity than for those with less‐stigmatized conditions like cardiomyopathy (Fino et al., 2026). The medical students were also more likely to attribute obesity to individual behavioural causes than genetic causes. Such experimental findings highlight how a patient's medical condition or weight status impacts medical students' assumptions and beliefs about patients. Further experimental research, extending to student midwives and dentists, is currently needed.

To address these gaps in the literature, the current research aimed to examine the impact of weight stigma on attitudes towards treating patients; predicted advice given to patients; and predicted time spent with patients in midwifery students (Study 1) and dental students (Study 2).

### Open science statement

Both studies were pre‐registered on the Open Science Framework (OSF) [Study 1: https://osf.io/nfzbv; Study 2: https://osf.io/ukbts]. Both studies were ethically approved via the University of Sheffield Ethics Review Procedure, as administered by the School of Psychology: Study 1 [Ref. 050377]; Study 2 [Ref. 039175].

## STUDY 1

### Aim and hypothesis

Study 1 aimed to examine midwifery students' attitudes towards and predicted time spent with a hypothetical patient that was described as either of healthy weight, overweight or obese. It was predicted that midwifery students would report more stigmatizing attitudes towards treating patients with overweight or obesity as compared to healthy‐weight patients (Hypothesis 1a) and would predict spending less time with patients with overweight and obesity compared to healthy‐weight patients (Hypothesis 1b).

### Methods

#### Design

The study employed an online experimental survey design, administered in Qualtrics (Provo, UT). It was a between‐participants design with weight status (healthy weight, overweight, obese) as the independent variable. Participants were randomly allocated to weight status conditions by Qualtrics. The primary dependent variables were the attitude towards treating the patient and the predicted time they would spend with the patient.

#### Participants

An a priori power analysis using G*Power 3.1.9.7 indicated that with *α* = .05, power = .80 and a medium effect size (*f* = .25; Faul et al., 2007), 159 participants were required for a one‐way ANOVA comparing the three weight status conditions. Participants were 18 years old or over and enrolled as midwifery students at a UK university. Recruitment occurred via social media, and advertisements circulated through the university midwifery and related departments. Participants were entered into a prize draw to win a £10 clothes voucher or an electronic gift voucher. Data were collected from November 2022 to February 2023.

#### Measures

##### Patient vignettes

Three patient case reports were developed for Study 1. The case reports were identical across conditions except for the patient's weight status (healthy weight, overweight or obese). Each case report described a 27‐year‐old pregnant woman at 12 weeks' gestation and included information about height, weight, body mass index (BMI), presenting concern (feeling nauseous most mornings), pregnancy history, medical history (no abnormalities) and health behaviours, including diet, physical activity and smoking. The vignette is provided in Supporting Materials.

##### Attitudes towards treating patients

Attitudes towards treating patients were assessed using an adapted version of the Attitudes Towards Treating Patients measure developed by Hebl and Xu (2001). The original 9‐point Likert scale was replaced with a 100‐point visual analogue scale from 0 [*not at all*] to 100 [*extremely*] to increase response sensitivity. Three of the original items assessing the seriousness of the patient's health problem, the amount of patient contact and the personal desire to help the patient were removed. References to ‘medical advice’ were modified to ‘midwifery advice’. The final measure included 10 items assessing perceptions of the patient's health, self‐care, self‐discipline, anticipated compliance with advice, perceived need for psychological counselling and overall positivity towards the patient (e.g., *‘How healthy would you judge the patient to be?’*). The internal consistency of the scale was acceptable for midwifery students (Cronbach's *α* = .66).

##### Predicted time spent

In line with previous research (Hebl & Xu, 2001), midwifery students were asked to predict the amount of time they would spend with the patient during a routine appointment. Time spent was assessed using a single item: *‘How much time would you spend with the patient for this regular check‐up appointment?’* Responses were recorded as a continuous measure (in minutes), with higher values indicating greater anticipated time spent with the patient.

##### Manipulation check

To assess whether participants had attended to the patient vignette, participants were asked to recall the patient's most recent healthcare appointment and the patient's main presenting concern from the case description.

##### Participant characteristics

Participants provided information on demographic characteristics, including age, gender, course enrolment, level of study (undergraduate or postgraduate) and ethnicity. Self‐perceived weight status was assessed with the item: ‘*How would you classify your own weight?*’ (underweight, healthy weight, overweight, obese). Additionally, participants reported the amount of formal obesity‐related education they had received during their professional training using the item: *‘As part of your midwifery training, how many hours of obesity education have you received?*’ Responses were recorded as a continuous variable.

#### Procedure

After providing informed consent, participants completed demographic questions and were screened for eligibility. Eligible participants were randomly allocated to one of three patient vignettes in which the patient was labelled as having a healthy weight, overweight or obesity. Participants then completed the attitude measure, predicted the amount of time they would spend with the patient and the advice they would give to the patient.^1^ Participants also responded to manipulation check questions to ensure participants had read the vignette. Finally, participants reported the number of hours of obesity‐related training they had received and indicated their perceived weight status (underweight, healthy weight, overweight or obese). Finally, participants were debriefed.

#### Data management and analysis

Participants were excluded if they failed to complete all of the key dependent variables. For the attitude measure, participants' data were only included in the relevant analysis if they had completed at least 80% of the items. Participants who failed both manipulation checks were excluded from analysis.^2^

All statistical analyses were conducted using IBM SPSS Statistics 29.0.0.0. Statistical significance was set at *p* < .05.

Baseline participant characteristics such as age, gender, ethnicity, self‐reported weight and years of training were compared across experimental conditions using one‐way ANOVAs with Bonferroni‐adjusted post hoc comparisons for continuous variables and Chi‐squared tests for categorical variables to assess the effectiveness of randomisation. As no significant differences were observed between conditions, these variables were not controlled for in subsequent analyses.

Two between‐participants one‐way ANOVAs were conducted to examine the effects of patient weight status (healthy weight, overweight, obese) on attitudes towards treating patients and predicted time spent with the patient. Bonferroni‐adjusted post hoc comparisons were used to explore significant main effects.

### Results

#### Participants' characteristics

A total of 471 participants accessed the survey. 281 participants remained after data cleaning. Exclusion was due to failure to start the study after giving consent (*n* = 83), failure to complete all the dependent variable measures (*n* = 102) or incorrect responses to both manipulation checks (*n* = 5). The mean age of the sample was 29.12 years (*SD* = 7.9 years). Most participants were White (95%), female (99%) and undergraduate students (93%). Further details are provided in Table 1.

**TABLE 1:** Study 1 participant characteristics in the healthy, overweight and obese conditions.

	Total (*n* = 281)	Healthy weight (*n* = 91)	Overweight (*n* = 95)	Obese (*n* = 95)
Age	29.12 years (*SD* = 7.9 years)	29.33 years (*SD* = 7.9 years)	29.43 years (*SD* = 8.5 years)	28.61 years (*SD* = 7.3 years)
Gender				
Male	0	0	0	0
Female	277 (99%)	90 (99%)	94 (99%)	93 (98%)
Other	3 (1%)	1 (1%)	0	2 (2%)
Prefer not to answer	1 (.4%)	0	1 (1%)	
Ethnicity				
White	268 (95%)	88 (97%)	90 (96%)	90 (96%)
Mixed	6 (2%)	1 (1%)	3 (3%)	2 (21%)
Asian or Asian British	2 (1%)	1 (1%)	1 (1%)	–
Black, African, Caribbean or Black British	3 (1%)	1 (1%)	0	2 (2%)
Prefer not to say	0	0	0	0
Other	1 (1%)	0	0	1 (1%)
Missing	1 (1%)	–	1 (1%)	I
Level of study				
Undergraduate	261 (93%)	85 (93%)	86 (91%)	90 (95%)
Postgraduate	16 (6%)	4 (4%)	7 (7%)	5 (5%)
Missing	4 (1%)	2 (2%)	2 (2%)	0
Self‐reported weight				
Obese	49 (17%)	16 (18%)	16 (17%)	17 (18%)
Overweight	93 (33%)	27 (30%)	26 (27%)	40 (42%)
Healthy weight	110 (39%)	39 (43%)	39 (41%)	32 (34%)
Missing	29 (10%)	9 (10%)	14 (15%)	6 (6%)
Obesity training				
None	68 (24%)	25 (28%)	24 (25%)	19 (20%)
Less than 1 h	43 (15%)	14 (15%)	15 (16%)	14 (14.7%)
1–2 h	65 (23%)	21 (23%)	18 (19%)	26 (27%)
3–5 h	50 (18%)	11 (12%)	17 (18%)	22 (23%)
6–10 h	16 (6%)	7 (8%)	4 (4%)	5 (5%)
11–15 h	3 (1%)	1 (1%)	0	2 (2%)
16+ h	7 (3%)	3 (3%)	3 (3%)	1 (1%)
Missing	29 (10%)	9 (10%)	14 (15%)	6 (6%)

#### Attitude towards treating patients

There was a significant main effect of patient weight status on attitudes towards treating patients, *F*(2, 278) = 3.06, *p =* .049, *η* ^2^ = .02. Bonferroni‐adjusted post hoc comparisons indicated that more stigmatizing attitudes were held towards overweight patients compared to healthy‐weight patients (mean difference = 35.97, *p* = .042). Attitudes did not significantly differ between healthy‐weight and obese patients (*p* = .678), nor between overweight and obese patients (*p =* .614). See Table 2 for descriptive statistics.

**TABLE 2:** Study 1 descriptive statistics for attitude and time spent.

Measure	Healthy weight	Overweight	Obese
Attitudes	16.7 (8.9)^a^ (*n* = 91)	20.30 (10.15)^b^ (*n* = 95)	18.47 (10.51)^ab^ (*n* = 95)
Predicted time spent (min)	20.83 (6.46) (*n* = 85)	22.98 (7.44) (*n* = 84)	21.98 (7.76) (*n* = 89)

*Note*: Values are means (standard deviations in parentheses). Means sharing the same superscript letter do not differ significantly at *p* < .05 (Bonferroni‐adjusted). Attitudes towards treating patients are scored such that a higher score indicates more stigmatizing attitudes.

##### Time spent

There was no significant effect of patient weight status on predicted time spent with the patient, *F*(2, 255) = 1.87, *p* = .157, *η* ^2^ = .014. See Table 2 for descriptive statistics.

### Discussion

Study 1's experimental study examined student midwives' attitudes towards treating patients and whether these attitudes were associated with the predicted time spent with patients of different weight statuses. In this experimental study, midwifery students demonstrated more stigmatizing attitudes towards patients with overweight compared to patients described as having a healthy weight. Interestingly, there was no difference in the stigmatizing attitude between patients with healthy weight and obesity. These findings are similar to the experimental study by Niemann et al. (2025) which found that medical students did not show a difference in their implicit bias towards pregnant patients with healthy weight (BMI; 23 kg/m^2^) and obesity (BMI; 33 kg/m^2^). However, unlike our study, Niemann et al., did not have a third category of patients with overweight (BMI; 29.9 kg/m^2^). More stigmatizing attitudes towards patients with overweight compared to a healthy weight may be interpreted through the attribution‐value model (Crandall et al., 2001), which proposes that prejudice is stronger towards groups perceived as responsible for their condition. In this case, individuals with overweight may be seen as having greater personal control over their weight (e.g., ‘lack of willpower’) than people with obesity, evoking stronger negative attitudes. Although much of the existing literature has focused on comparisons between healthy‐weight and overweight individuals (Chambliss et al., 2004; Persky & Eccleston, 2011; Sabin et al., 2012), there is little research directly comparing stigma towards overweight versus obese individuals. An alternative explanation for these results is that midwifery training and practice place a strong emphasis on health behaviours, risk management and personal responsibility during pregnancy (Hildingsson et al., 2016; World Health Organization, 2021). Overweight individuals may therefore be perceived as more personally responsible for their weight, as it is often viewed as modifiable through behavioural change. In contrast, obesity may be more readily attributed to less controllable factors, such as metabolic, endocrine or medical conditions, which could reduce perceived blame. Despite more negative attitudes towards the patient with overweight compared to a healthy weight, midwifery students' predicted time spent with patients did not significantly differ between conditions.

## STUDY 2

### Aim and hypothesis

Study 2 aimed to compare dental students' attitudes towards and predicted time spent with a hypothetical patient that was described as either of healthy weight, overweight or obese. Study 2 also compared the predicted advice provided between conditions, which was categorized as dental health versus lifestyle‐related (e.g., dietary behaviours, exercising). Study 2 also manipulated patient gender (female, male) in addition to patient weight status. Though the gender of the patients did not significantly impact physicians' attitude towards patients in Hebl and Xu (2001), other studies have reported that women tend to have a greater risk of experiencing weight bias and stigmatization (Puhl et al., 2008). A group of non‐dentist students was also included as a comparator to explore whether stigma was greater in dental students compared to students from other non‐dental disciplines. Non‐dental students were included as a comparator group, consistent with previous research (e.g., Rathbone et al., 2023). This comparison group provided a broader reference point for determining whether observed weight‐stigmatizing attitudes are specific to dental students or reflect attitudes that may also be present among students generally.

Finally, we also examined whether student dentists' beliefs about obesity predicted stigmatizing attitudes towards patients. It was predicted that dental students would report more stigmatizing attitudes towards treating patients with overweight or obesity as compared to healthy‐weight patients (Hypothesis 2a), would predict spending less time with patients with overweight and obesity compared to healthy‐weight patients (Hypothesis 2b), and would be more likely to provide advice related to lifestyle to patients with overweight and obesity compared to healthy‐weight patients (Hypothesis 2c). No hypothesis was developed concerning whether dental and non‐dental students would differ in their attitudes towards treating patients and in the advice provided to patients of different weight statuses. An exploratory (not pre‐registered) hypothesis predicted that dental and non‐dental students who more strongly attribute obesity to individual factors (behavioural or psychological) would report more negative attitudes towards patients (Hypothesis 2d).^3^

### Methods

#### Design

Similar to Study 1, an experimental design was used. However, Study 2 had a 3 (weight status: healthy weight, overweight, obese) × 2 (patient gender: female, male) × 2 (participant group: dental students, non‐dental students) between‐participants design, and additional dependent variables were assessed. Specifically, advice provided, categorized as dental health or lifestyle‐related (with the latter indicative of weight stigma), and beliefs about the causes of obesity.

#### Participants

An a priori power analysis using G*Power 3.1.9.2 indicated that with *α* = .05, power = .80 and a small effect size (*f* = .15), 864 participants were required to detect the 3‐way interaction between patients' weight status, patient gender and participant group on attitudes and predicted time spent with patients. The final sample was 528, which was insufficient to test the 3‐way interaction. However, a power analysis using G*Power 3.1.9.7 indicated that with *α* = .05, power = .80 and a small effect size (*f* = .15), 390 participants were sufficient to test the 2‐way interaction between patients' weight status and participant group on attitudes and predicted time spent with patients. Accordingly, as per the contingency plans in the pre‐registered protocol, patient gender was not included as a factor in the analyses.

Participants were 18 years old or older and enrolled at a UK university. Dental students with fewer than three years of undergraduate dental education were excluded from participation following advice from a dental expert that a holistic level of dental expertise is obtained after 3 years of study. Non‐dental students could be enrolled on any course. Students were recruited via university email distribution lists, social media (e.g., Twitter, Facebook, LinkedIn), the online participant recruitment platform Prolific, and posters displayed in dental schools or public university spaces (e.g., libraries, student unions). Dental students were entered into a prize draw to win a £50 shopping voucher, while non‐dental students were entered into a prize draw to win a £25 shopping voucher. Participants recruited via Prolific received automatic payment through the platform (£1.00) and were not eligible to enter a prize draw. The data were collected from February 2020 to April 2023.

#### Measures

Participants' characteristics, attitudes towards treating patients (Cronbach's *α* = .65 for dental students; *α* = .77 for non‐dental students), predicted time spent, and manipulation checks were the same as measured in Study 1.

##### Patient vignettes

Six patient case reports were developed based on previous research by Hebl and Xu (2001) and adapted for relevance to dentistry. The case reports were identical across conditions, such that only patient gender (male, female) and weight status (healthy weight, overweight, obese) were manipulated. Each case report described a 31‐year‐old patient and included information on the patient's height, weight, gender, marital status, occupation, blood pressure and medical history. The vignette described the patient as never having had their teeth cleaned, having some anxiety about dental work and their last dental visit was more than 10 years ago for wisdom tooth extraction. The patient presented with anterior tooth discolouration, including concern about the whiteness of their teeth and visible dark and yellow discolouration. This dental issue was selected because tooth discolouration can result from a range of potential causes, providing an opportunity to examine whether advice given to patients was more focused on dietary behaviours when the patient was described as overweight or obese compared with healthy weight. The patient's health behaviours included brushing twice daily, occasional flossing, mouthwash use, never smoking, drinking approximately 12 units of alcohol per week, and daily tea or coffee consumption. The case reports were informed by existing educational materials used for dental students (Crest + Oral‐B, n.d.) and were reviewed by a dental expert from the University of Sheffield to ensure clinical appropriateness and realism. The vignettes are in the Supporting Materials.

##### Beliefs about the causes of obesity

Beliefs about the causes of obesity were assessed using a well‐established 11‐item questionnaire developed by Foster et al. (2003). This measure assesses the extent to which individuals believe a range of behavioural, physiological and psychological factors contribute to obesity. To reflect more recent conceptualisations of obesity, three additional items were included assessing the perceived importance of inexpensive unhealthy foods, advertising for unhealthy foods and food addiction, consistent with prior research (Puhl et al., 2015). In line with previous research, items were categorized into four sub‐scales: behavioural (physical inactivity, overeating, high‐fat diet, restaurant eating and repeated dieting), psychological (poor nutritional knowledge, psychological problems, lack of willpower and food addiction), physiological (metabolic defects, endocrine disorders and genetic factors), and environmental causes (inexpensive unhealthy foods and advertising for unhealthy foods) of obesity (Puhl et al., 2015). Behavioural and psychological sub‐scales were conceptualized as individual‐level (and potentially more stigmatizing) attributions, whereas physiological and environmental sub‐scales were conceptualized as non‐individual attributions that locate responsibility beyond the individual. Participants rated each item on a 100‐point scale ranging from 0 (*not at all important*) to 100 (*extremely important*). A mean score was calculated for each subscale such that higher scores indicated stronger endorsement of that factor as a cause of obesity.

##### Predicted advice provided

Participants were asked to provide written responses to the open‐ended question: *‘What advice would you give to the patient?’* This item was included to assess the type and focus of advice offered across patient weight conditions. The advice was categorized as dental health (e.g., brushing, scaling and polishing) versus lifestyle‐related (e.g., dietary behaviours related to weight, exercising) by the first author.

#### Procedure

After providing informed consent, participants provided their demographic information and were screened for eligibility. Eligible participants were randomly assigned to one of six patient vignettes varying by weight status (healthy, overweight or obese) and gender (male or female). All participants then completed the attitude measure and answered manipulation check questions to confirm they had read the case report. Dental students then reported the advice they would give to the patient, predicted the amount of time they would spend with the patient, reported their beliefs about the causes of obesity, the amount of obesity training (if any) they had completed, and responded to questions regarding their willingness to offer obesity intervention to patients and which strategy they preferred.^4^ Non‐dental students reported the advice they would give to the patient and reported their beliefs about the causes of obesity. Finally, participants were debriefed.

#### Data management and analysis

Data management and the procedure were identical to Study 1. However, two 3 × 2 between‐participants factorial ANOVAs were conducted to examine the effects of patient weight status (healthy weight, overweight, obese) and participant group (dental students, non‐dental students) on attitudes towards treating patients and predicted time spent with the patient. Bonferroni‐adjusted post hoc comparisons were used to explore significant main effects where relevant. Significant interaction effects were explored using Bonferroni‐adjusted independent‐samples *t*‐tests (to compare dental and non‐dental students) and a one‐way ANOVA followed by Bonferroni‐adjusted post hoc comparisons (to examine the effects of patient weight status in dental students only). Due to an oversight, only the former was pre‐registered. While the pre‐registration specified that chi‐square analyses would be conducted to examine whether predicted advice differed between dental and non‐dental students, logistic regression was deemed more appropriate, as it allows for the prediction of advice type while accounting for student category and other relevant predictors simultaneously. Finally, a multiple regression analysis (enter method) was conducted to examine whether beliefs about the causes of obesity (personal, genetic and environmental) predicted mean attitude scores.

### Results

#### Participant characteristics

A total of 483 dental students and 332 non‐dental students accessed the survey. 528 participants (255 dental students and 273 non‐dental students) remained after data cleaning. Non‐dental participants were studying health and biological sciences (19%), with the remaining 81% enrolled across a range of other non‐health‐related disciplines. Further details are provided in the supplementary material.

For dental students, exclusion was due to failure to start the study after giving consent (*n* = 107), failure to complete all the dependent variable measures (*n* = 112) or incorrect responses to both manipulation checks (*n* = 9). For non‐dental students, exclusion was due to failure to start the study after giving consent (*n* = 8), failure to complete all the dependent variable measures (*n* = 67) or incorrect responses to both manipulation checks (*n* = 2). The mean age of the sample was 22.82 years (*SD* = 3.96 years). Most participants were White (67%), female (50%), and undergraduate students (93%). Further details are provided in Table 3.

**TABLE 3:** Study 2 participant characteristics in the healthy, overweight and obese conditions.

	Total	Healthy weight (*n* = 89)	Dental students (*n* = 255)			Non‐dental students (*n* = 273)		
			Overweight (*n* = 85)	Obese (*n* = 81)	Total	Healthy weight (*n* = 93)	Overweight (*n* = 91)	Obese (*n* = 89)
Age	22.82 years (*SD =* 3.9 years)	22.06 years (*SD* = 2.6 years)	22.78 years (*SD* = 5.7 years)	23.26 years (*SD* = 2.8 years)	22.34 years (*SD* = 5.6 years)	22.10 years (*SD* = 4.4 years)	23.07 years (*SD* = 6.6 years)	21.85 years (*SD* = 5.4 years)
Gender								
Male	103 (40%)	35 (39%)	36 (42%)	32 (40%)	61 (22%)	25 (27%)	22 (24%)	14 (16%)
Female	127 (50%)	48 (54%)	39 (46%)	40 (49%)	206 (76%)	68 (73%)	66 (73%)	72 (81%)
Others	1 (.4%)	0	1 (1%)	0	5 (2%)	0	3 (3%)	2 (2%)
Prefer not to answer	0	0	0	0	1 (.4%)	0	0	1 (1%)
Missing	22 (9%)	6 (7%)	9 (11%)	9 (11%)	0	0	0	0
Ethnicity								
White	170 (67%)	60 (67%)	54 (64%)	56 (70%)	209 (77%)	69 (74%)	68 (75%)	72 (81%)
Mixed	11 (4%)	5 (6%)	3 (4%)	3 (4%)	8 (3%)	6 (7%)	2 (2%)	0
Asian or Asian British	36 (14%)	12 (14%)	15 (18%)	9 (11%)	41 (15%)	12 (4%)	18 (20%)	11 (12%)
Black, African, Caribbean or Black British	4 (2%)	2 (2%)	1 (1%)	1 (1%)	9 (3%)	4 (4%)	2 (2%)	3 (3%)
Prefer not to say	6 (2%)	2 (2%)	1 (1%)	3	6 (1%)	2 (2%)	1 (1%)	3 (3%)
Other	5 (2%)	3 (3%)	2 (2%)	0	0	0	0	0
Missing	23 (9%)	5 (6%)	9 (11%)	9 (11%)	–	–	–	–
Level of study								
Undergraduates	237 (93%)	84 (94%)	80 (94%)	73 (90%)	213 (78%)	75 (81%)	72 (79%)	66 (74%)
Postgraduates	11 (4%)	4 (5%)	4 (5%)	3 (4%)	60 (22%)	18 (19%)	19 (21%)	23 (26%)
Missing	7 (3%)	1 (1%)	1 (1%)	5 (6%)	0	0	0	0
Own weight status								
Obese	4 (2%)	2 (2%)	1 (1%)	1 (1%)	–	–	–	–
Overweight	21 (8%)	6 (7%)	5 (6%)	10 (12%)	–	–	–	–
Healthy weight	206 (81%)	75 (84%)	69 (81%)	62 (77%)	–	–	–	–
Underweight	5 (2%)	0	3 (4%)	2 (3%)	–	–	–	–
Missing	19 (8%)	6 (7%)	7 (8%)	6 (7%)	–	–	–	–
Obesity Training								
None	24 (9%)	10 (11%)	6 (7%)	8 (10%)	–	–	–	–
Less than 1 h	39 (15%)	17 (19%)	5 (6%)	17 (21%)	–	–	–	–
1–2 h	53 (21%)	14 (16%)	23 (27%)	16 (20%)	–	–	–	–
3–5 h	66 (26%)	22 (25%)	26 (31%)	18 (22%)	–	–	–	–
6–10 h	27 (11%)	10 (11%)	7 (8%)	10 (12%)	–	–	–	–
11–15 h	18 (7%)	9 (10%)	4 (5%)	5 (6%)	–	–	–	–
16+ h	11 (4%)	4 (5%)	7 (8.2%)	0	–	–	–	–
Missing	17 (7%)	3 (3%)	7 (8%)	7 (9%)	–	–	–	–

*Note*: Own weight status and obesity training were not measured for non‐dental students.

#### Attitude towards treating patients

See Table 4 for descriptive statistics. There was a significant main effect of patient weight status on attitudes towards treating patients, *F*(2, 522) = 25.58, *p* < .001, *ηp* ^2^ = .089. Post‐hoc Bonferroni comparisons revealed that more stigmatizing attitudes were held towards overweight (mean difference = 8.13, *p* < .001) and obese patients (mean difference = 7.57, *p* < .001) compared to healthy‐weight patients.

**TABLE 4:** Study 2 descriptive statistics for attitude and time spent, as a function of participant group and patient weight status.

Dependent variable	Condition	Dental students	Non‐dental students
Attitude	Healthy weight	37.62 (11.92)^a^ (*n* = 89)	36.69 (12.90)^a^ (*n* = 93)
	Overweight	45.01 (10.70)^b^ (*n* = 85)	45.51 (12.38)^b^ (*n* = 91)
	Obese	42.07 (11.17)^b+^ (*n* = 81)	47.13 (12.01)^b^ (*n* = 89)
	Total	41.31 (8.98) (*n* = 255)	43.03 (13.23) (*n* = 273)
Predicted time spent (min)	Healthy weight	50.58 (37.47) (*n* = 88)	–
	Overweight	55.97 (44.82) (*n* = 79)	–
	Obese	51.11 (34.89)	–
	Total	52.48 (39.14) (*n* = 79)	–

*Note*: Values are means (standard deviations in parentheses). Means sharing the same superscript letter do not differ significantly at *p* < .05 (Bonferroni‐adjusted). ^+^Indicates a significant difference between dental and non‐dental students for the same patient weight category. Attitudes towards treating patients are scored such that a higher score indicates more stigmatizing attitudes. Predicted time spent was not measured for non‐dental students.

Attitudes towards overweight and obese patients did not differ significantly (mean difference = .55, *p =* 1.000). There was no significant main effect of participant group on attitudes towards treating patients, *F*(1, 522) = 2.21, *p = .138*, *ηp* ^2^ = .004.

However, there was a significant interaction between patient weight status and participant group, *F*(2, 522) = 2.99, *p* = .05, *ηp* ^2^ = .011. This was explored in two ways. To compare dental and non‐dental students, follow‐up independent‐samples *t*‐tests were conducted, indicating that dental students reported significantly less stigmatizing attitudes towards obese patients compared to non‐dental students (mean difference = −5.06), *t*(168) = −2.83, *p* = .003. In contrast, there were no significant differences between dental and non‐dental students in attitudes towards overweight patients (mean difference = −.5), *t*(174) = −.29, *p* = .387 or healthy‐weight patients (mean difference = .93), *t*(180) = .51, *p* = .307. To examine effects of patient weight status in dental students, a one‐way ANOVA was conducted on dental students only, revealing a significant main effect of patient weight status, *F*(2, 251) = 9.50, *p* < .001, *η* ^2^ = .07. Bonferroni‐adjusted post hoc comparisons showed that attitudes towards treating overweight patients were significantly more stigmatizing than towards healthy‐weight patients (mean difference = 7.44, *p* < .001). Attitudes towards treating obese patients were also significantly more stigmatizing than towards healthy‐weight patients (mean difference = 4.50, *p* = .031). There was no significant difference in attitudes towards treating overweight vs. obese patients (mean difference = 2.94, *p* = .287).

#### Predicted time spent

There was no significant effect of patient weight status on predicted time spent with the patient, *F*(2, 243) = .47, *p* = .629, *η* ^2^ = .004. See Table 4 for descriptive statistics.

#### Advice

A binary logistic regression examined whether participant group and patient weight status predicted the likelihood of providing stigmatizing advice. The model indicated a significant effect of participant group, with dental students showing substantially higher odds of providing lifestyle‐related advice than non‐dental students, *B* = 3.07, SE = .37, Wald = 68.33, *p* < .001 OR = 21.49. There was also a significant effect of patient weight status, Wald *χ* ^2^(2) = 6.90, *p* = .032. Compared to healthy‐weight patients, participants were more likely to provide lifestyle‐related advice to overweight patients (*p* = .017) and obese patients (*p* = .025). See Table 5 for descriptive statistics.

**TABLE 5:** Study 2 descriptive statistics for advice given to patients by dental and non‐dental students, as a function of patient weight status.

	Dental students		Non‐dental students	
	Dental‐advice (%)	Lifestyle (e.g, eating patterns, exercise) (%)	Dental‐advice (%)	Lifestyle (e.g, eating patterns, exercise) (%)
Healthy weight	72.5	27.5	97.8	2.2
Overweight	49.2	50.8	96.8	3.25
Obese	53.8	46.2	95.5	4.5
Total	58.8	41.2	96.3	3.3

#### Beliefs about obesity

A multiple linear regression examined whether beliefs about obesity as behavioural, psychological, environmental or physiological predicted attitudes towards patients among dental and non‐dental students. For dental students, beliefs about behavioural (*β* = .15, *p* = .078), psychological (*β* = −.15, *p* = .100), physiological (*β* = .14, *p* = .079) and environmental causes of obesity (*β* = .06, *p* = .437) did not significantly predict attitudes towards treating patients. Similarly, for non‐dental students, beliefs about behavioural (*β* = −.08, *p* = .339), psychological (*β* = .16, *p* = .070), physiological (*β* = −.09, *p* = .139) and environmental causes of obesity (*β* = .12, *p* = .088) were not significant predictors of attitudes towards treating patients. See Table 6 for descriptive statistics.

**TABLE 6:** Study 2 descriptive statistics for beliefs about obesity in dental and non‐dental students, as a function of patient weight status.

	Dental students				Non‐dental student			
	Healthy weight (*n* = 86)	Overweight (*n* = 80)	Obese (*n* = 79)	Total (*n* = 245)	Healthy weight (*n* = 91)	Overweight (*n* = 89)	Obese (*n* = 88)	Total (*n* = 268)
Behavioural	62.63 (16.69)	64.14 (11.78)	61.35 (13.7)	62.71 (13.11)	75.78 (16.36)	70.06 (15.31)	71.83 (14.94)	72.44 (15.85)
Psychological	69.88 (15.91)	64.15 (16.46)	66.48 (15.38)	66.91 (16.04)	66.05 (16.29)	61.49 (16.26)	62.29 (15.77)	63.23 (16.16)
Physiological	44.80 (16.82)	43.71 (12.72)	45.61 (15.44)	44.70 (15.67)	52.77 (19.41)	54.31 (19.83)	51.40 (20.31)	52.69 (19.90)
Environmental	68.65 (17.68)	65.71 (19.23)	67.29 (20.82)	67.25 (19.20)	64.34 (18.54)	61.22 (21.04)	62.78 (19.39)	62.74 (19.62)

### Discussion

Similar to study 1, dental and non‐dental students had more stigmatizing attitudes towards patients who were overweight compared to healthy‐weight patients, and there was no difference in the predicted time spent. However, unlike study 1, dental students had more stigmatizing attitudes towards both patients with overweight and obesity. One possible explanation for this difference between studies is that dentists may be less likely to differentiate between overweight and obesity, as body weight is not a central focus of dental care and they receive limited formal education regarding obesity (Awan et al., 2016). Instead, higher body weight may be viewed more broadly as a general health risk, resulting in more uniformly negative attitudes towards both overweight and obese individuals.

Furthermore, results indicated that patient weight status significantly affected the likelihood of receiving lifestyle‐related advice. Compared with healthy‐weight patients, overweight and obese patients were more than twice as likely to be the target of lifestyle‐related recommendations. Additionally, dental students were significantly more likely than non‐dental students to provide lifestyle‐related advice (i.e., to eat healthy, exercise and lose weight), with the odds being over 20 times higher. This did not appear to be due to differences in attitudes between dental and non‐dental students; there was no significant effect of the participant group on attitudes towards treating patients.

## GENERAL DISCUSSION

The current research comprises two experimental studies examining healthcare students' attitudes towards patients of different weight statuses and whether these attitudes were associated with predicted time spent with patients and the advice provided to them. Study 1 assessed midwifery students, while Study 2 assessed dental and non‐dental students. The findings align with previous research reporting stigmatizing attitudes in healthcare professionals' attitudes towards people with overweight (e.g., Elboim‐Gabyzon et al., 2020; George et al., 2019; Miller Jr et al., 2013; Swift et al., 2013) and extend these findings to trainee midwives and dentists.

The current research also found no evidence that patient weight status impacted midwifery and dental students' predicted time spent with patients. This finding contrasts with a previous experimental study in which physicians predicted spending less time with overweight than healthy‐weight patients (Hebl & Xu, 2001). The discrepancy may reflect differences in healthcare systems. The present study was conducted in the UK, where publicly funded care involves fixed appointment lengths, potentially constraining predicted consultation time. In contrast, Hebl and Xu's US‐based study took place in a paid healthcare system that may allow greater flexibility in consultation length. Additionally, the discrepant findings could be explained by social changes over time, including a significant increase in the prevalence of overweight and obesity (NCD Risk Factor Collaboration, 2024), which may have normalized higher‐weight patients in healthcare settings. Notably, the two earlier studies that assessed time spent with patients were conducted over 20 years ago (Bertakis & Azari, 2005; Hebl & Xu, 2001), and as such, changes in societal attitudes and healthcare training may have influenced more recent findings.

Overall, our results suggest that explicit weight bias may not influence all aspects of clinical care equally. Although students reported explicitly negative attitudes towards higher‐weight patients, these attitudes did not influence predicted time spent with patients in either dental or midwifery students but were associated with stigmatizing advice in dental students. It is possible that procedural expectations and professional standards override personal attitudes when students estimate the amount of time they would spend with patients. In contrast, weight bias may be more likely to emerge in qualitative aspects of care, such as the type and framing of advice provided. It is also possible that stigma manifests more strongly in the quality of patient–provider interactions rather than their duration. These findings highlight the need for future research to examine how weight stigma influences different dimensions of healthcare delivery, particularly the interpersonal and communicative aspects of care.

The findings have important implications for healthcare professionals. Current forecasts predict that by 2050, 50% of the world's population will be overweight or obese (GBD 2021 Adult BMI Collaborators, 2025). Given the current findings and the broader literature demonstrating the impacts of weight stigma on mental (Emmer et al., 2020) and physical health (Romano et al., 2023; Sehrawat et al., 2026), it is critical that we develop effective interventions to reduce stigmatizing attitudes among healthcare professionals. Researchers have recommended a person‐centred approach, which shifts the focus from weight to health, adopting size and weight‐inclusive practices, building empathy for patients of all sizes, using weight‐inclusive language and reassessing clinical guidelines and policies that rely on BMI as a primary criterion (Bailey et al., 2020; Levinson et al., 2024; Ulian et al., 2018). Moreover, there is a clear need for curricula targeted at trainees in healthcare professions that explicitly address the topic of weight stigma, educate about obesity, promote health at every size and emphasize compassionate communication. There is evidence that healthcare knowledge of obesity (Nickel et al., 2019) and using weight‐neutral terminology (Puhl, 2020) reduce weight biases.

Interventions are particularly important within midwifery, given the substantial changes in body weight that can occur during pregnancy and the high global prevalence of overweight and obesity, which affects approximately 48% of pregnancies (see systematic review by Kent et al., 2024). As such, weight stigma can be a common experience for pregnant women (Mulherin et al., 2013). Weight stigma during pregnancy has been associated with a range of adverse experiences and outcomes, including gestational diabetes, anxiety and concerns about being overmedicated during antenatal care, perceived pressure to undergo caesarean delivery because of weight and avoidance of or disengagement from professional maternity and postpartum care (DeJoy et al., 2016; Nagpal, Tomiyama, & Incollingo Rodriguez, 2021; Ward & McPhail, 2019). Furthermore, maternal health is closely linked to child wellbeing (see systematic review by Mudiyanselage et al., 2024). These findings highlight the importance of addressing weight stigma within midwifery education. Equipping midwifery students to recognize and challenge weight‐based assumptions may help ensure that women receive respectful, non‐stigmatizing and equitable care throughout pregnancy and the postpartum period, aligning with public health priorities such as the National Health Service's Three‐Year Delivery Plan for Maternity and Neonatal Services (NHS England, 2023).

Similarly, weight stigma training is important for dental students. Weight stigma can contribute to delayed or avoided healthcare (Aldrich & Hackley, 2010; Gnade et al., 2020; Graham et al., 2022), including avoidance of dental services (Malik et al., 2021). A scoping review has also identified weight‐stigmatizing attitudes among dental professionals (Malik et al., 2023). This is particularly relevant given the growing prevalence of overweight and obesity globally (World Health Organization, 2025) and evidence linking overweight and obesity with poorer oral health. For example, children living with obesity have been reported to have higher rates of dental caries (Public Health England, 2019b), and adult obesity has been associated with poorer oral hygiene (Alshehri et al., 2020; Kim et al., 2020). Poor oral health is also associated with, but not limited to, cardiovascular disease (Hopkins et al., 2024) and cancer (Mathur et al., 2019). Children and adults with overweight and obesity therefore need dental care environments that are compassionate and non‐stigmatizing to support attendance at regular check‐ups, which are important for maintaining oral and overall health.

Furthermore, there is consensus among the health care professionals, including dentists, for integrating weight‐management services into their practice to contribute to early identification and prevention of further weight gain (Large et al., 2024; Public Health England, 2019a), thereby potentially reducing pressure on other NHS services. However, such opportunities depend on patients feeling comfortable engaging with dental professionals about their weight and health. Further confirming the need to develop effective interventions to support non‐stigmatizing practice among dental students.

The current studies have several limitations. Firstly, the studies relied on self‐report measures, which may underestimate weight stigma due to social desirability bias (Oppenheim, 1992). Future research may explore using implicit measures of stigma, which may capture attitudes that participants may be unwilling to report (e.g., see Greenwald & Banaji, 1995). Second, the inability to fully examine patient gender effects in Study 2 due to sample size constraints may have obscured intersectional patterns of stigma. Prior research shows that weight stigma is gendered, with women experiencing greater bias, which may amplify stigma at the intersection of weight and gender (Puhl et al., 2008). Finally, the presence of weight bias during training does not necessarily indicate that students will maintain negative attitudes after graduation (Obara et al., 2018). Nevertheless, existing evidence suggests that such attitudes may persist into professional practice within healthcare settings (Puhl et al., 2016; Sabin et al., 2012). Future research should adopt longitudinal approaches to examine how weight‐related attitudes develop, change or persist over time as students transition into professional healthcare roles.

## CONCLUSION

The present study provides novel experimental evidence that weight stigma is present in trainee healthcare professionals and can impact the advice given to patients. Both midwifery and dental students reported negative attitudes towards treating higher‐weight patients, though these attitudes did not translate into differences in predicted time spent with patients. Notably, dental students provided more stigmatizing advice, whereas explicit beliefs about obesity had no impact on negative attitudes towards treating patients. Addressing weight stigma in healthcare training is critical for promoting equitable and compassionate patient care.

## AUTHOR CONTRIBUTIONS

**Ankita Sehrawat:** Methodology; investigation; formal analysis; writing – original draft; writing – review and editing. **Ayse Aslan:** Conceptualization; methodology; investigation. **Eleanor L. Aldam:** Conceptualization; methodology; investigation. **Chantelle Wood:** Writing – review and editing; supervision. **Nicola J. Buckland:** Conceptualization; methodology; writing – review and editing; supervision.

## CONFLICT OF INTEREST STATEMENT

The authors declare no conflicts of interest.

## ETHICS STATEMENT

Ethics approval was obtained from the ethics committee of the School of Psychology, University of Sheffield, for both studies: Midwifery [050377] and Dental and Non‐dental students [039175].

## PROTOCOL REGISTRATIONS ON OPEN SCIENCE FRAMEWORK

**Study 1**: https://osf.io/nfzbv.

**Study 2:** https://osf.io/ukbts.

## Supporting information


**Table S1.** Number and percentage of non‐dental students included in Study 2.

## Data Availability

The data that support the findings of this study are available from the corresponding author upon reasonable request.

## Footnotes

^1^
The pre‐registration also included a plan to measure and analyse the effects of weight stigma on predicted advice given to patients. However, this variable was later excluded from analysis and is not reported here. This is because standard perinatal guidance routinely includes lifestyle recommendations, making it difficult to isolate the effects of stigma on advice.

^2^
Sensitivity analyses were also conducted by repeating the main analyses after including participants who provided incorrect responses to both the manipulation check questions. The interpretation of these analyses is the same as the main analyses. Therefore, for parsimony, the results from these analyses are included in the Supplemental materials.

^3^
While the pre‐registration also included hypotheses evaluating potential differences between dental and non‐dental students on beliefs about obesity, this was beyond the scope of the current paper, and instead formed part of a student dissertation.

^4^
While the pre‐registration also included additional questions regarding obesity interventions, these were beyond the scope of the current paper and instead formed part of a student dissertation.
