# Supplementary material for: The impact of patient weight status on healthcare students' attitudes: Two experimental studies in midwifery and dental students
Source: Br J Health Psychol. 2026 Oct 28;31(4):e70116. doi: 10.1111/bjhp.70116 (PMC13717204; doi:10.1111/bjhp.70116)
Supplement: Supplementary file 1 [file BJHP-31-0-s001.docx]

**The Impact of Patient Weight Status on Healthcare Students’ Attitudes: Two Experimental Studies in Midwifery and Dental Students**

**Study 1: Patient vignette**

**Patient Profile:** Patient is a 27-year-old woman who has a healthy weight [BMI: 22.9; height: 1.65m; weight: 62.4kg]/overweight [BMI: 29.9; height: 1.65m; weight: 81.4kg]/ obese [BMI: 35; height: 1.65m; weight: 95.4kg]. Patient is 12 weeks pregnant.

**Main complaint:** Patient reports feeling nauseous most mornings.

**Check-up history:** Patient's 12-week scan showed no concerns.

**Medical history:** No family history of pregnancy-related complications. Currently takes no medications. Health records show the last blood pressure was recorded at 115/70. Urine dipstick shows no abnormalities.

**Health behaviours:** Patient practices meditation twice a day. Drinks water regularly throughout the day. Never smoked.

**Study 2: Patient vignette**

**Male patient case report**

**Patient profile:** Patient is a 31-year-old man who has a healthy weight [height: 1.78 m (5′ 10″); weight: 72.6 kg (11 st 6 lb)]; is overweight [height: 1.78 m (5′ 10″); weight: 95.3 kg (15 st 0 lb)]; is obese [height: 1.78 m (5′ 10″); weight: 113.5 kg (17 st 12 lb)]. Patient reports he has never had his teeth cleaned and has some anxiety about dental work.

**Main complaint:** Patient reports a lot of concern about his anterior teeth not being very white. Two of his teeth show darkness and a yellow tint.

**Dental history:** His last dental visit was over ten years ago when he had a wisdom tooth extracted.

**Medical history:** No family history of chronic health conditions. Currently takes no medications. Health records show his last blood pressure reading was recorded at 138/89.

**Health behaviours:** Patient uses a manual toothbrush twice a day. Flosses when he remembers to and occasionally uses mouthwash. Reports drinking 12 units of alcohol per week (beer and wine). Drinks tea or coffee daily. Never smoked.

**Other information:** marital status not reported. Works for a marketing company.

**Female patient case report**

**Patient profile:** Patient is a 31-year-old woman who has a healthy weight [height: 1.65 m (5′ 5″); weight: 62.4 kg (9 st 12 lb)]; is overweight [height: 1.65 m (5′ 5″); weight: 81.7 kg (12 st 12 lb)]; is obese [height: 1.65 m (5′ 5″); weight: 95.5 kg (15 st 1 lb)]. Patient reports she has never had her teeth cleaned and has some anxiety about dental work.

**Main complaint:** Patient reports a lot of concern about her anterior teeth not being very white. Two of her teeth show darkness and a yellow tint.

**Dental history:** Her last dental visit was over ten years ago when she had a wisdom tooth extracted.

**Medical history:** No family history of chronic health conditions. Currently takes no medications. Health records show last blood pressure was recorded at 138/89.

**Health behaviours:** Patient uses a manual toothbrush twice a day. Flosses when she remembers to and occasionally uses mouthwash. Reports drinking 12 units of alcohol per week (beer and wine). Drinks tea or coffee daily. Never smoked.

**Other information:** marital status not reported. Works for a marketing company.

Table S1. Number and percentage of non-dental students included in Study 2.

| **Discipline** | **Students (*n*)** | **%** |
| --- | --- | --- |
| Health / Biological Sciences | 53 | 19.4% |
| Psychology | 37 | 13.6% |
| Business / Management / Finance | 27 | 9.9% |
| Arts / Design / Creative | 22 | 8.1% |
| Computing / Computer Science | 17 | 6.2% |
| Engineering | 14 | 5.1% |
| History / Archaeology | 13 | 4.8% |
| English / Languages | 13 | 4.8% |
| Law | 12 | 4.4% |
| Criminology / Policing | 11 | 4.0% |
| Medicine | 10 | 3.7% |
| Mathematics / Physics | 9 | 3.3% |
| Social Sciences | 8 | 2.9% |
| Education | 7 | 2.6% |
| Politics / International Relations | 5 | 1.8% |
| Geography / Planning | 3 | 1.1% |
| Forensic Science | 1 | 0.4% |
| Missing | 4 | 1.5% |
| Other/unclear | 6 | 2.2% |
| PhD / Research | 1 | 0.4% |
| Total | 273 | 100% |
